# Pilot-scale production of xylo-oligosaccharides and fermentable sugars from *Miscanthus* using steam explosion pretreatment

**DOI:** 10.1016/j.biortech.2019.122285

**Published:** 2020-01

**Authors:** Rakesh Bhatia, Ana Winters, David N. Bryant, Maurice Bosch, John Clifton-Brown, David Leak, Joe Gallagher

**Affiliations:** a Institute of Biological, Environmental and Rural Sciences (IBERS), Aberystwyth University, Plas Gogerddan, Aberystwyth SY23 3EE, UK; b Department of Biology & Biochemistry, University of Bath, Bath BA2 7AY, UK

**Keywords:** *Miscanthus*, Steam explosion, Xylo-oligosaccharides, Biofuels, Biorefining

## Abstract

This study investigated pilot-scale production of xylo-oligosaccharides (XOS) and fermentable sugars from *Miscanthus* using steam explosion (SE) pretreatment. SE conditions (200 °C; 15 bar; 10 min) led to XOS yields up to 52 % (w/w of initial xylan) in the hydrolysate. Liquid chromatography-mass spectrometry demonstrated that the solubilised XOS contained bound acetyl- and hydroxycinnamate residues, physicochemical properties known for high prebiotic effects and anti-oxidant activity in nutraceutical foods. Enzymatic hydrolysis of XOS-rich hydrolysate with commercial *endo*-xylanases resulted in xylobiose yields of 380 to 500 g/kg of initial xylan in the biomass after only 4 h, equivalent to ~74 to 90 % conversion of XOS into xylobiose. Fermentable glucose yields from enzymatic hydrolysis of solid residues were 8 to 9-fold higher than for untreated material. In view of an integrated biorefinery, we demonstrate the potential for efficient utilisation of *Miscanthus* for the production of renewable sources, including biochemicals and biofuels.

## Highlights

• ~50 % (w/w) of initial xylan in biomass recovered as XOS in the liquid fraction.
• Xylobiose yields of up to 500 g/kg of initial xylan using commercial enzymes.
• ~75 % conversion of initial xylan in biomass to XOS.
• Up to 70 % hydrolysis of glucan from residual solids to fermentable glucose.

## Graphical abstract

**Figure:**
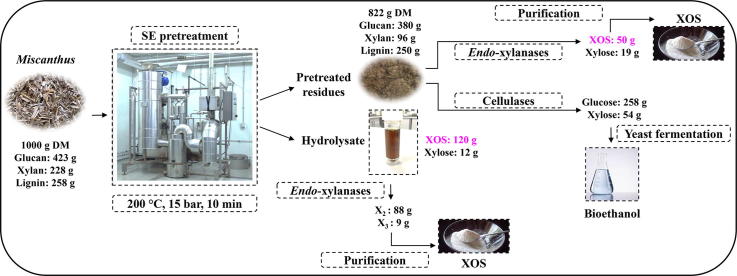

## 1 Introduction

The European Union (EU) is committed to a reduction of >40 % in greenhouse gas (GHG) emissions and a 25 % increase in the total European transportation fuels from biofuels by 2030 to meet and potentially exceed the objectives of the Paris Agreement for long-term decarbonisation of the EU energy system. Such goals are driven in part by the transition from fossil-based fuels to carbon-neutral plant biomass-based renewable energy sources (European Commission, 2018). The dedicated biomass crop *Miscanthus*, closely related to the leading biofuel crops maize and sugarcane, is a high yielding C_4_ rhizomatous perennial grass that combines high photosynthetic, nutrient and water efficiency and is well adapted to a wide range of climates and soil types with good environmental credentials (Clifton-Brown et al., 2017). Field trials have demonstrated the potential for *Miscanthus* to produce up to 17 t/ha of harvestable dry matter (DM) biomass per year in the UK (Hastings et al., 2009). *Miscanthus* is an established biomass crop for co-firing in power stations within Europe (Terravesta, 2019) but also represents an abundant and renewable feedstock for refining into biofuels, bio-based materials and chemicals. Hence, more readily up-scalable high biomass yielding and seed-based hybrids of *Miscanthus* are being developed and are expected to be market-ready by around 2022, to contribute more to renewable energy and GHG mitigation targets, and for expansion of the European bio-economy (Clifton-Brown et al., 2019).

Pretreatment is a crucial processing step to increase the porosity and reduce the recalcitrance of biomass to deconstruction for the production of biofuels and biochemicals (Chaturvedi and Verma, 2013). Depending on the type and severity of pretreatment, these processes can be costly and energy-intensive. Moreover, they can have distinct effects on the separation of the major components of lignocellulosic biomass, i.e. glucan (~25–40 %), xylan (~25–50 %) and lignin (~10–30 %) as well as result in sugar losses or by-products inhibitory to enzymes and microbial fermentation (Menon and Rao, 2012). Steam explosion (SE) is generally recognised as a scalable, cost-effective pretreatment technique with a low environmental impact (Chen and Liu, 2015). Industries such as the Brazilian biotechnology company GranBio have commercialised a SE pretreatment technology platform for converting sugarcane bagasse into biofuels and biochemicals (GranBio, 2014). During SE pretreatment, biomass is saturated with steam at high temperatures (160–240 °C) and pressures (7–48 bar) for several minutes (5–15 min) and then exposed to atmospheric pressure making the material undergo an explosive decompression (Chen and Liu, 2015). Hydronium ions generated from the dissociation of water under high temperature and pressure act as a weak acid to cleave the acetyl- and uronyl-groups attached to the xylan backbone. This promotes the formation of acetic and uronic acid, which in turn catalyses (auto-hydrolysis) removal of xylan with the limited dissolution of glucan, while lignin undergoes fragmentation and recondensation reactions (Pu et al., 2015).

Xylo-oligosaccharides (XOS) are emergent value-added compounds due to their prebiotic properties for use in food and pharmaceuticals as well as a variety of other applications (Amorim et al., 2019, Kumar et al., 2012). The worldwide prebiotic market is expected to grow from ~3.6 € billion in 2017 to ~6.6 € billion by 2023 (MarketsandMarkets, 2018). A variety of XOS with a degree of polymerisation (DP) ranging from 2 to 10 xylose units can be recovered subject to SE process conditions and biomass substrates (Kumar et al., 2012). XOS are a hydrolysis product of xylan, which generally consists of a xylose backbone decorated with various substitutions including arabinofuranosyl, acetyl-groups, and ester-linked phenolic acids including ferulic and *p*-coumaric acid. In addition, the solubilisation and removal of xylan from lignocellulosic biomass via SE could help optimise biofuel production costs, as the pretreatment also causes disruption of the cellulose microfibrils and increases the accessibility of the glucan fraction to enzymes during hydrolysis (Alvira et al., 2010, Brodeur et al., 2011). Moreover, XOS have been shown to act as potent inhibitors of cellulase activity in converting glucan into fermentable sugars (Qing et al., 2010). Several xylan-rich agro-residues have been assessed for XOS production via SE pretreatment, including sugarcane bagasse (Carvalho et al., 2018, Silva et al., 2018, Silveira et al., 2018, Zhang et al., 2018), sugarcane straw (Oliveira et al., 2013), corn stover (Liu et al., 2013), and wheat straw (Álvarez et al., 2017, Huang et al., 2017). There have been limited efforts though to employ *Miscanthus* for the production of value-added XOS compounds.

This study evaluated the generation of XOS and fermentable sugars from *Miscanthus* hybrids involving SE pretreatment. Several SE process variables such as temperature, residence time and biomass particle sizes were tested using a pilot-scale SE unit, and their effects on sugar recovery and formation of fermentation inhibitors in SE hydrolysates were considered as evaluation criteria for pretreatment efficiency. The yields and DP of XOS were quantified, and the structural features of XOS were identified. Enzymatic digestion of SE hydrolysate with commercial *endo*-xylanases resulted in the depolymerisation of XOS into low-DP (2–3) XOS, while the SE pretreated solids showed improvements in enzymatically digested glucose yields for potential bioethanol co-production. We highlight a potential integrated biorefinery process strategy for the efficient utilisation of the dedicated biomass crop *Miscanthus*.

## 2 Materials and methods

### 2.1 Raw material

*Mx2779*, also known as GNT-14, is a leading novel seed-based interspecies hybrid (*Miscanthus sinensis × M. sacchariflorus*) bred in Aberystwyth in 2013. *Mx2779* entered pre-commercial seed production trials in Catania, Italy in 2016 because earlier multi-location screens in Germany and UK showed it was both high biomass yielding (DM yield in year three ~14 t/ha) and more drought tolerant than standard *Miscanthus × giganteus* (*Mxg*). Three replicate plots of *Mx2779* progeny each comprising forty-eight individuals, planted at 1.5 m spacings, were planted along with *Mxg* controls on 6 June 2014 at a location near Aberystwyth. Above-ground biomass (leaves and stem) following senescence of the two *Miscanthus* hybrids (*Mx2779* and *Mxg*) were harvested in March 2016 at Aberystwyth. Biomass material was hammer chipped into an average size of ~10 to 30 mm, dried at 45 °C per technical report NREL/TP-510–42620 (Hames et al., 2008) and stored in dumpy bags until the raw material was pretreated by SE. A representative portion of the biomass material was also hammer milled and sieved into an average size of 0.18–0.85 mm (−20/+80 mesh) for SE pretreatment, as biomass particle size represents one of the most important factors affecting SE performance. Biomass moisture content was determined per technical report NREL/TP-510-42621 (Sluiter et al., 2008a) and biomass was used as received for SE pretreatment.

### 2.2 Steam explosion of *Miscanthus*

*Miscanthus* biomass (0.25 kg) was suspended at a water/solid ratio of 10:1 (g/g) and then imbibed with deionised water at various conditions (0 h, 2 h at 70 °C, 24 h at 25 °C, 24 h at 70 °C) to evaluate XOS recoveries post SE pretreatment. Before SE pretreatment, the liquid and solid components were separated by draining the excess liquid through a muslin cloth, and aliquots of the liquid filtrate were retained to determine sugar content (monomeric and oligomeric) per technical report NREL/TP-510-42623 (Sluiter et al., 2008b). A maximum of 0.2 % xylose, 2.4 % glucose, 0.5 % arabinose and 0.9 % galactose as well as 0.3 % XOS and 0.5 % GOS (w/w % of initial DM sugar) were quantified and solubilised into this liquid filtrate. The strained biomass fraction (~75 % moisture content) was loaded into the Cambi pilot-scale 30 L SE rig (Cambi, Norway) and pretreatment was performed at reaction temperatures of 180 °C (9 bar), 200 °C (15 bar), 210 °C (20 bar) and 225 °C (25 bar) with short residence times at either 5 min, 10 min or 15 min, desirable for a scale-up process. For each pretreatment condition, at least two explosions were carried out. The severity factor (SF) of the individual SE pretreatments was calculated according to the equation,

$$SF=Log10\left[t\;x\;e\frac{T-100}{14.75}\right]$$

where t is the residence time (min), T is the temperature (°C) of pretreatment, 100 is the base temperature, i.e. 100 °C and 14.75 is the arbitrary constant which is an empirical parameter related with activation energy and temperature from first-order kinetics (Carvalheiro et al., 2009). In this study, SF was in the range of 3.4 to 4.7. It should be noted that the performance of SE pretreatment is also highly dependent on biomass particle sizes and moisture content, which were not considered in calculating SF.

Following SE pretreatment, the material was recovered in 10 L containers and cooled to room temperature. 0.5 L of deionised water was added to the pretreated slurry to recover water-soluble carbohydrates from the solids, and the slurry was homogenised and strained through a muslin cloth to separate the liquid and solid fractions. An aliquot of the liquid hydrolysate post-SE was retained for analysis of sugars as well as by-products and degradation products. Another aliquot of the hydrolysate was subjected to end-hydrolysis with 4 % H_2_SO_4_ per technical report NREL/TP-510-42623 (Sluiter et al., 2008b) for total sugar content and composition (monosaccharides and oligosaccharides). The pretreated solid residues were stored in labelled sealable polythene bags at -20 °C until further use. Biomass recovered (%) was estimated as DM solids remaining after pretreatment per 100 g of DM raw material. Compositional analysis of untreated and SE pretreated solids (washed with deionised water to remove hydrolysate) was determined per technical reports NREL/TP-510-42618 (Sluiter et al., 2012), NREL/TP-510-48825 (Sluiter and Sluiter, 2011) and NREL/TP-510-42627 (Sluiter et al., 2008c).

### 2.3 Oligosaccharide analysis

XOS and gluco-oligosaccharides (GOS) were analysed simultaneously by High-Performance Anion Exchange Chromatography (Thermofischer ICS-5000) coupled with Pulse Amperometric Detector (HPAEC-PAD), using the Dionex CarboPac PA200 guard (3 × 50 mm) and analytical (3 × 250 mm) columns. An injection volume of 25 μL of each sample was analysed by HPAEC-PAD with the column temperature set at 30 °C, and an eluent flow rate of 0.3 mL/min. Analytical reagent grade sodium hydroxide (50 % w/w) and sodium acetate were obtained from Sigma-Aldrich. The HPAEC-PAD elution program for XOS and GOS quantification was as follows: from 0 to 9 min 100 % A (0.1 M NaOH) and 0 % B (0.5 M NaOAc/0.1 M NaOH), from 9.1 to 32 min 92 % A (0.1 M NaOH) and 8 % B (0.5 M NaOAc/0.1 M NaOH), from 32.1 to 46 min 50 % A (0.1 M NaOH) and 50 % B (0.5 M NaOAc/0.1 M NaOH) and from 46.1 to 56 min 100 % A (0.1 M NaOH) and 0 % B (0.5 M NaOAc/0.1 M NaOH). XOS standards (X_2_) xylobiose, (X_3_) xylotriose, (X_4_) xylotetraose and (X_5_) xylopentaose as well as GOS standards (G_2_) cellobiose, (G_3_) cellotriose and (G_4_) cellotetraose (Megazyme) were run as calibration standards using serial dilution concentration ranges of 20 μg/mL, 10 μg/mL, 5 μg/mL, 2.5 μg/mL and 1.25 μg/mL.

### 2.4 Monomeric sugars, by-products and degradation products analysis

Monosaccharides were quantified with a Thermo Scientific™ Dionex™ HPAEC-PAD ICS-5000 system using the Dionex CarboPac SA10 guard (4 × 50 mm) and analytical (4 × 250 mm) columns at 45 °C, and 1 mM KOH as eluent, with an eluent flow rate of 1.5 mL/min and 25 μL injection volume. Glucose, xylose, arabinose, galactose, mannose, fructose, sucrose, cellobiose and fucose were run as calibration standards using serial dilution concentration ranges of 20 μg/mL, 10 μg/mL, 5 μg/mL, 2.5 μg/mL and 1.25 μg/mL. Monosaccharide chromatograms were analysed and processed using the Chromeleon™ 7.2 Chromatography Data System (CDS) software.

Organic acids were analysed by HPLC equipped with a refractive index detector using 5 mM H_2_SO_4_ mobile phase at 55 °C and a flow rate of 0.6 mL/min through the Aminex HPX-87H column (Bio-Rad) per technical report NREL/TP-510-42623. Organic acid chromatograms were analysed and processed using the Chromeleon™ 5.0 Chromatography Data System (CDS) software. The degradation products and by-products formed in the hydrolysate from SE pretreatment are presented as a proportion of initial DM biomass, as variations in SE hydrolysate volume occur, often not highlighted in SE pretreatment studies.

### 2.5 Phenolics quantification and physicochemistry of XOS by LC-MS

Partial purification of hydrolysates was carried out by solid-phase extraction (SPE) using Sep-Pak C18 cartridges (Waters Ltd, Elstree, UK) as described by Hauck et al., (2014) and dried using a heated vacuum centrifuge at 60 °C. Dried samples were dissolved in 0.5 mL 70 % methanol before analysis. Samples were analysed by reverse-phase HPLC equipped with photodiode array detection and coupled with an electrospray ionisation tandem mass spectrometry (HPLC-PDA-ESI/MSN) on a Thermo Finnigan system (Thermo Electron Corp, Waltham, MA, USA). Separation of compounds was carried out on a Waters C18 Nova-Pak column (3.9 × 100 mm, particle size 4 µm) at 30 °C with a flow rate of 1 mL/min and injection volume of 10 µL. The mobile phase consisted of water with 0.1 % formic and acid (A) and methanol with 1 % formic acid (B) with B increasing from 5 to 65 % in 30 min. Eluting compounds were detected with a Finnigan PDA Plus detector between 240 and 400 nm and a Finnigan LTQ linear ion trap with an ESI source. MS parameters were as follows: sheath gas 30, auxiliary gas 15 and sweep gas zero (arbitrary units), spray voltage −4.0 kV in negative and 4.8 kV in positive ionisation mode, capillary temperature 320 °C, capillary voltage −1.0 and 45 V, respectively, tube lens voltage −68 and 110 V, respectively, and normalised collision energy (CE) typically 35 %. Data were acquired and processed using Thermo ScientificTM XcaliburTM software, and phenolic compounds were quantified using response factors for coumaric, ferulic acid, vanillin and syringaldehyde using PDA data. Relative quantification of substituted XOS was carried out by estimating the area under the curve for selected *m*/*z* chromatograms in negative mode.

### 2.6 Enzymatic hydrolysis of hydrolysate and SE pretreated solids

Commercial *endo*-xylanases NS22083 and NS22002 (Novozymes) were used to produce XOS with a DP range of 2–3 xylose units from the hydrolysate. *Endo*-xylanase activity was measured using arabinoxylan (wheat flour; medium viscosity ~30 cSt, 12.5 mg/mL Megazyme) as substrate as described by the Megazyme Somogyi reducing sugar assay. One unit of enzyme activity is the amount of enzyme required to release one µmole of reducing sugar equivalents (as xylose by the Somogyi reducing-sugar method) from arabinoxylan per minute under standard assay conditions (40 °C and pH 4.7). The measured enzyme activity for NS22083 and NS22002 was 3610 U/mL and 690 U/mL, respectively. The SE hydrolysate was first centrifuged before enzymatic hydrolysis to remove suspended solids. 1 mL of the SE hydrolysate was then mixed with 42 µL of 1 M sodium citrate buffer (pH 5) and with 5.6 µL of 5 % sodium azide. The total volume in each tube was brought to 1.4 mL with Milli-Q water after the *endo*-xylanase NS22083, or NS22002 was added at a dosage of 36 U/mL of hydrolysate (Huang et al., 2017). Doubling of enzyme load was used to further ensure that enzyme concentration was non-limiting in the assay. Samples were loaded in a shaker set at 50 °C (200 rpm) and withdrawn after different time points (0, 4, 24 and 48 h). The hydrolysis reaction was stopped by boiling the samples at 100 °C for 10 min and samples were analysed for XOS by HPAEC.

Enzymatic hydrolysis of pretreated solid residues was based on the technical report NREL/TP-5100-63351 (Resch et al., 2015) using Cellic® CTec2 (Novozymes) at a high dosage of 30 % w/w (g enzyme/g glucan) as outlined per the Novozymes Cellic® CTec2 application sheet for hydrolysis of lignocellulosic materials. Samples were loaded in a shaker set at 50 °C (200 rpm) and withdrawn after 72 h. The hydrolysis reaction was stopped by boiling the samples at 100 °C for 10 min and samples were analysed for glucose yields by HPAEC.

## 3 Results and discussion

### 3.1 Biomass composition of high yielding *Miscanthus* hybrids

The biomass composition of the two *Miscanthus* hybrids, *Mx2779* and *Mxg*, used in this study are highlighted in Table 1. The carbohydrate constituents accounted for ~60 % of dry matter (DM) and were mainly composed of glucan (~37 %) and xylan (~20 %). The Klason lignin (~21–23 %), extractives (~7–10 %) and ash (~3–5 %) content also varied depending on the *Miscanthus* hybrid. Acetyl content (~4 %) was relatively higher in both *Miscanthus* hybrids compared with that of other lignocellulosic residues from grasses such as corn stover (~3 %) and rice straw (~2 %) (Harun et al., 2013, Selig et al., 2009). A higher acetyl-group content has previously been demonstrated advantageous for efficient auto-hydrolysis of sugarcane bagasse and thus for the production of XOS (Zhang et al., 2018). The biomass composition of *Mxg* used in this study was comparable to other previously reported studies (Chen et al., 2014, Ji et al., 2015), though it may differ depending on plant growth conditions including climate, soil and use of fertiliser (Lewandowski et al., 2016).

**Table 1:** Cell wall composition of two high biomass yielding *Miscanthus* hybrids on an as-received basis (a) and extractives free basis (b). Data are means ± standard error (n ≥ 3).

a									
	As-received basis (w/w % of DM solids)								
*Miscanthus*	Glucan	Xylan	Arabinan	Galactan	Acid insoluble lignin	Acid soluble lignin	Acetyl	Ash	Extractives
*Mx2779*	36.4 ± 0.4	19.5 ± 0.2	2.8 ± 0.1	1.1 ± 0.0	21.0 ± 0.4	1.1 ± 0.0	3.9 ± 0.1	4.6 ± 0.0	9.5 ± 0.0
*Mxg*	38.6 ± 0.2	20.4 ± 0.1	2.8 ± 0.0	0.7 ± 0.0	22.8 ± 0.1	0.9 ± 0.0	4.0 ± 0.0	3.1 ± 0.0	6.7 ± 0.2

b							
	Extractives free (w/w % of DM solids)						
*Miscanthus*	Glucan	Xylan	Arabinan	Galactan	Acid insoluble lignin	Acid soluble lignin	Acetyl
*Mx2779*	42.3 ± 0.5	22.8 ± 0.3	3.3 ± 0.1	1.3 ± 0.0	24.5 ± 0.5	1.3 ± 0.0	4.6 ± 0.1
*Mxg*	42.8 ± 0.2	22.7 ± 0.1	3.1 ± 0.0	0.8 ± 0.0	25.3 ± 0.1	0.9 ± 0.0	4.4 ± 0.0

### 3.2 Steam explosion pretreatment for XOS production

Key factors affecting XOS production by SE pretreatment were investigated in this study, including temperature, residence time, as well as biomass particle sizes. The choice and range of conditions employed were based on previously published SE pretreatment literature (Chen et al., 2014, Walker et al., 2018, Zhang et al., 2018). Preliminary experiments for the *Miscanthus* hybrids indicated that neither 180 °C (~10 % w/w of initial xylan), nor 210 °C (~15 % w/w of initial xylan) and 225 °C (~3 % w/w of initial xylan) were useful for hydrolysis of xylan into XOS. Other studies have similarly shown 200 °C as the optimal temperature for high XOS yields (Álvarez et al., 2017, Chen et al., 2014, Oliveira et al., 2013, Zhang et al., 2018). It is noteworthy that chipped biomass particles (~10 to 30 mm) lead to higher XOS yields (55 % w/w of initial xylan) when compared with the yields (49 % w/w) of milled and smaller biomass particles (0.18–0.85 mm) at the same SE pretreatment conditions (200 °C; 15 bar; 10 min). This trend is likely due to the lower bulk density and higher porosity of *Miscanthus* biomass at larger particle size allowing for more efficient steam penetration and pretreatment reaction, than that of smaller biomass particle size (Liu et al., 2013). These results infer that *Miscanthus* does not necessarily require extensive mechanical processing into smaller particle sizes to reduce biomass recalcitrance or to eliminate mass and heat transfer limitations during SE pretreatment. Hence, processing with larger *Miscanthus* biomass particle size is recommended, as this could significantly help lower energy consumption and operating costs as well as reduce carbohydrate losses associated with grinding and milling of biomass (Liu et al., 2013). In additional preliminary experiments, the amount of XOS increased from 20 to 55 % (w/w of initial xylan) when increasing the SE residence time from 5 min to 10 min and then decreased to 35 % (w/w) as residence time was increased to 15 min. Employing a deionised H_2_O pre-washing step (2 h at 70 °C, 24 h at 25 °C, or 24 h at 70 °C) prior to SE pretreatment (200 °C; 15 bar; 10 min) did not drastically improve XOS recovery, which remained stable between 51 and 55 % (w/w of initial xylan). Hence, chipped biomass material (~10 to 30 mm) along with the SE conditions (200 °C; 15 bar; 10 min) were selected for further analysis in this study.

**Fig. 1:**
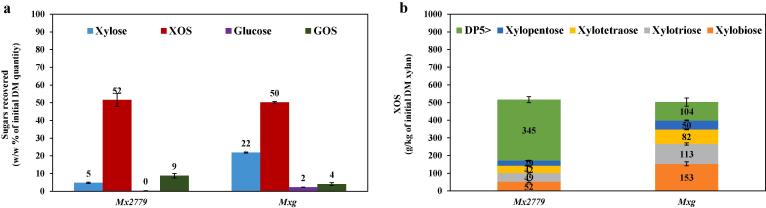
Sugars recovered (a) and XOS with different DP produced (b) from SE pretreated (200 °C; 15 bar; 10 min) *Miscanthus* without enzymatic hydrolysis. Data are means ± standard error (n ≥ 2). DM, dry matter; XOS, xylo-oligosaccharides; GOS, gluco-oligosaccharides.

The SE parameters (200 °C; 15 bar; 10 min) for *Mx2779* resulted in XOS yields up to 52 % (w/w) and a low yield of xylose ~5 % (w/w) (Fig. 1a). While for *Mxg*, SE parameters (200 °C; 15 bar; 10 min) gave XOS yields up to 50 % (w/w) and a relatively high concentration of xylose ~22 % (w/w) (Fig. 1a), the latter i.e. xylose monomers usually regarded as an impurity and unfavourable for the production of valuable XOS compounds. In order to satisfy commercial XOS compound standards of 80 % purity, the concentration of xylose in the XOS-rich hydrolysate can be reduced for example by fermenting xylose into ethanol (Huang et al., 2019). The ~60 to 70 % (w/w) recovered as XOS and xylose from SE pretreated *Miscanthus* (Fig. 1a) is in alignment with the content of 60 to 70 % easily hydrolysable xylan reported by Chen et al., 2014. This is likely due to the presence of xylan side groups decreasing the adsorption of xylan to glucan considerably, whereas the remaining ~30–40 % (w/w of initial xylan) is likely to represent unsubstituted linear xylan which has been shown to favour adsorption to glucan (Kabel et al., 2007). Although Chen et al., 2014 reported XOS yields up to ~69 % (w/w of initial xylan) from *Mxg* under conditions of 200 °C for 5 min, the authors reported their findings based on an auto-hydrolysis step using a lab-scale batch mini-reactor system. These findings would imply that XOS production from *Miscanthus* for commercialisation cannot be solely based on the XOS yields obtained in experimental laboratory trials and hence the need for pilot-scale studies. Nonetheless, the similar XOS yields (~50 % w/w) but different xylose yields (5–22 % w/w) under the same SE conditions for the two *Miscanthus* hybrids (Fig. 1a), are possibly related to distinctive structural properties of their plant cell walls. Likewise, HPAEC analysis provided information regarding the concentration and DP distribution of XOS produced from SE pretreatment of *Miscanthus* hybrids, with 52 vs 153 g/kg xylobiose, 49 vs 113 g/kg xylotriose, 42 vs 82 g/kg xylotetraose, 29 vs 50 g/kg xylopentaose, and 345 vs 104 g/kg DP5 > of initial xylan when comparing *Mx2779* with *Mxg* respectively (Fig. 1b). These results further suggest distinctive biomass features of the *Miscanthus* hybrids that may affect xylan hydrolysis into XOS and thus promote the concept of genetically engineering or breeding *Miscanthus* hybrids suited for specific end-use applications and biorefining products (da Costa et al., 2019).

### 3.3 Composition of steam explosion pretreated solids, XOS and hydrolysate

The composition of *Miscanthus* solids after SE pretreatment was found to be in the range of ~40 to 45 % (w/w) for glucan, ~5 to 7 % (w/w) for xylan, ~1 to 2 % (w/w) for acetyl-residues and ~24 to 27 % (w/w) for lignin (Table 2a). Other minor components, including arabinan and galactan, were also detected in the residual solids (Table 2a). As a reference, Chen et al., 2014 reported recovery of ~66 % (w/w) glucan, ~7 % (w/w) xylan and ~19 % (w/w) lignin in pretreated *Mxg* solids by auto-hydrolysis (200 °C; 5 min). As expected, SE pretreatment enriched glucan and lignin content compared to the untreated *Miscanthus* biomass (Table 1 and Table 2a), while xylan and acetyl concentrations decreased as these latter fractions are released during the auto-hydrolysis step into the hydrolysate. Glucan recovery was high (~88–95 %) whereas xylan recovery was low (~21–27 %) (Table 2a), likely because xylan is less crystalline and more thermally labile than glucan.

**Table 2:** Composition of pretreated solids (a), and oligosaccharides (b) as well as degradation products and by-products (c) in the hydrolysate from *Miscanthus* under SE pretreatment conditions (200 °C; 15 bar; 10 min). Data are means ± standard error (n ≥ 2).

a											
	Extractives free pretreated residual solids (w/w % of DM solids)								Carbohydrate Recovery (%)		
*Miscanthus*	Glucan	Xylan	Arabinan	Galactan	Acid insoluble lignin	Acid soluble lignin	Acetyl	Biomass recovered (w/w % DM solids)	Glucan	Xylan	Delignification (%)
*Mx2779*	40.4 ± 0.3	6.7 ± 0.1	0.4 ± 0.0	0.1 ± 0.0	26.0 ± 0.5	0.7 ± 0.0	1.6 ± 0.1	91.3 ± 1.7	87.6 ± 0.7	27.3 ± 0.6	4.1 ± 0.4
*Mxg*	46.8 ± 0.5	5.4 ± 0.1	0.3 ± 0.0	0.0 ± 0.0	23.2 ± 1.0	0.6 ± 0.0	1.2 ± 0.0	88.5 ± 4.9	96.7 ± 1.0	21.2 ± 0.2	19.6 ± 3.4

b					
	Composition of oligomers in hydrolysate (w/w % of initial DM solids)				
*Miscanthus*	Xylose	Arabinose	Glucose	Galactose	Acetic acid
*Mx2779*	10.1 ± 0.7	0.3 ± 0.0	3.2 ± 0.4	0.4 ± 0.0	0.8 ± 0.1
*Mxg*	10.3 ± 0.5	0.4 ± 0.1	1.6 ± 0.0	0.5 ± 0.0	1.4 ± 0.1

c														
By-products and degradation products in hydrolysate (g/kg of DM solids)														
*Miscanthus*	Biomass particle sizes (mm)	Pre-soaking with H_2_O	SE condition	SF	Hydrolysate pH	HMF	Furfural	Lactic acid	Formic acid	Acetic acid	Vanillin	Syringaldehyde	Coumaric acid	Ferulic acid
*Mx2779*	~10 to 30	No	200 °C; 15 bar; 10 min	3.9	4.3	0.9 ± 0.1	2.6 ± 0.2	5.0 ± 1.2	7.0 ± 0.9	18.6 ± 1.6	0.6 ± 0.1	0.4 ± 0.0	0.7 ± 0.0	0.4 ± 0.1
*Mxg*	~10 to 30	No	200 °C; 15 bar; 10 min	3.9	3.7	3.7 ± 0.3	4.4 ± 0.5	11.7 ± 2.3	6.5 ± 1.6	21.8 ± 1.8	1.1 ± 0.1	0.9 ± 0.1	1.3 ± 0.1	0.7 ± 0.1

Biomass recovered (%) = gram of DM residual solids recovered after SE pretreatment/100 g DM untreated biomass.

Component recovery (%) = (Component content in SE pretreated solids × biomass recovered)/Total component in untreated biomass.

Delignification (%) = 100 – Lignin recovery (%) in SE pretreated solids.

DM, dry matter; HMF, Hydroxymethylfurfural; SE, steam explosion; SF, severity factor.

The composition of oligomers in SE hydrolysate was mainly comprised of xylose (~10 % w/w), arabinose (~0.5 % w/w), galactose (~0.5 % w/w), acetic acid (~1 % w/w) and glucose (~2–3 % w/w) (Table 2b). LC-MS analysis further allowed for structural elucidation of differently substituted XOS, mainly acetylated, coumarylated, and feruloylated XOS obtained from SE hydrolysate. A variety of acetylated XOS ranging from DP 2 to 9 were identified in the mass spectra. Furthermore, LC-MS detected lower levels (~2-fold) of acetylated XOS in the hydrolysate for *Mx2779* compared with *Mxg* after SE pretreatment, which is in accordance with the amount of acetic acid quantified for the oligomers in the hydrolysate (Table 2b). The LC-MS data also indicated that the majority of the XOS (~84–89 %) contained two or more attached acetyl-groups. Additionally, a series of XOS substituted with *p*-coumaric and ferulic acids, inherent components of xylan–lignin complexes, were observed in the LC-MS mass spectra for the *Miscanthus* hybrids. These findings substantiate the prebiotic and antioxidant potential of XOS compounds, which are attributed to such substitutions as well as the DP of XOS (Singh et al., 2015).

In addition to assessing SE conditions for high xylan-to-XOS yield, characterising the composition of the XOS-rich hydrolysate for undesired monosaccharides and nonsaccharide compounds is important as these components may require removal, for instance via downstream purification steps, to obtain pure XOS. Different concentrations of by-products and sugar degradation products in the hydrolysate generated by SE conditions are highlighted in Table 2c. A broad range of compounds such as hydroxymethylfurfural (HMF) and furfural can be formed from the degradation of hexose and pentose sugars, respectively (Hu and Ragauskas, 2012), while acetic acid is produced by de-acetylation from xylan and formic acid is a product of HMF and furfural breakdown (Almeida et al., 2007). These products act as impurities and at elevated concentrations as inhibitory compounds to fermenting micro-organisms and thus can represent a major challenge for commercial production of bioethanol and other platform chemicals derived from lignocellulosic biomass (Zabed et al., 2016). Under the SE parameters which yielded the highest amount of XOS, *Mx2779* and *Mxg* contained very low concentrations of the degradation compounds HMF (~1–4 g/kg) and furfural (~3–4 g/kg) of DM solids (Table 2c), equivalent to about 0.1 to 0.2 g/L of HMF and 0.3 g/L of furfural. In addition, other degradation and by-products measured were lactic acid (~5–12 g/kg), formic acid (~7 g/kg) and acetic acid (~20 g/kg) (Table 2c), equivalent to ~0.6 g/L of lactic acid, ~0.6 g/L of formic acid and ~1.5 g/L of acetic acid. Moreover, very low concentrations of phenolic compounds were generated (Table 2c), such as *p*-coumaric and ferulic acid which are involved in crosslinking xylan and lignin in grasses (Carpita, 1996), and vanillin and syringaldehyde derived from the degradation of guaiacyl (G) and syringyl (S) units of lignin. Similar results were found for wheat straw and sugarcane bagasse using SE pretreatment (Álvarez et al., 2017, Huang et al., 2017, Silveira et al., 2018). The difference in pH values of the SE hydrolysate obtained from *Mx2779* (4.3) and *Mxg* (3.7) (Table 2c) are likely a result of the initial amount of extractives and ash in the biomass causing a buffering effect. When SE temperature and residence time was increased to more harsh conditions, i.e. beyond 200 °C and 10 min, a lower hydrolysate pH and greater amount of inhibitory products such as HMF (~5 g/kg), furfural (~5–8 g/kg) and acetic acid (~25 g/kg) were generated. Collectively, only minor degradative effects on glucan, lignin and xylan were exerted during SE pretreatment conditions (200 °C; 15 bar; 10 min), and the low concentrations of degradation and by-products obtained from SE pretreated *Miscanthus* indicate that a purification step for their removal prior to downstream enzymatic hydrolysis and fermentation may not be necessary.

### 3.4 Enzymatic hydrolysis of steam explosion hydrolysate

**Fig. 2:**
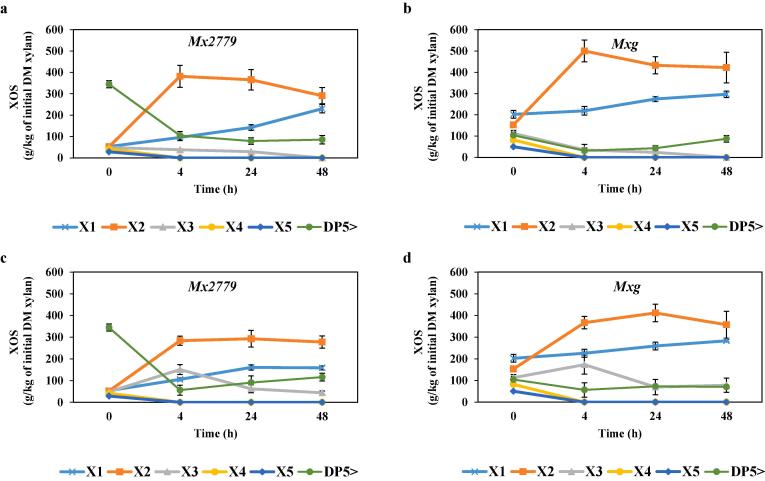
Enzymatic hydrolysis with *endo*-xylanases NS22083 (a and b) and NS22002 (c and d) of SE hydrolysate (200 °C; 15 bar; 10 min) from *Miscanthus*. Data are means ± standard error (n ≥ 2).

To further improve the selective release of xylobiose and xylotriose which represent the most valuable XOS compounds for health benefits (Amorim et al., 2019, Singh et al., 2015), enzymatic hydrolysis with commercial *endo*-xylanase preparations of the SE hydrolysate was performed. As shown in Fig. 2, high DP XOS was effectively cleaved by the commercially available Novozymes *endo*-xylanases NS22083 and NS22002 to fragments mainly of xylobiose and with the lower formation of xylotriose. Moreover, the XOS profile produced by the *endo*-xylanases were similar between the *Miscanthus* hybrids but yielding different amounts of predominantly xylose, xylobiose and xylotriose. For *Mx2779*, xylobiose increased from 52 to ~380 g/kg and from 52 to ~280 g/kg of initial xylan, while for *Mxg* xylobiose increased from 153 to ~500 g/kg and from 153 to ~370 g/kg of initial xylan using NS22083 and NS22002 respectively, within 4 h of enzymatic hydrolysis (Fig. 2). The proportion of xylobiose represents an increase of ~6-fold and ~3-fold for *Mx2779* and *Mxg*, respectively, when compared to the SE hydrolysate without *endo*-xylanase treatment. Alongside the increase in xylobiose after 4 h of enzymatic hydrolysis, XOS with DP > 5 decreased as these were hydrolysed to lower DP (2–3) XOS or xylose monomers (Fig. 2). In general, Novozymes *endo*-xylanase NS22002 (Fig. 2c and d) was more effective than NS22083 (Fig. 2a and b) for generating xylotriose within 4 h of hydrolysis. Further research should focus on evaluating the low-DP (2–3) XOS prebiotic activities and anti-oxidant properties for end-use applications, and the discovery of novel *endo*-xylanases could help tailor substituted XOS for superior health-promoting effects (Nordberg Karlsson et al., 2018).

### 3.5 Enzymatic hydrolysis of steam explosion pretreated solid residues

**Fig. 3:**
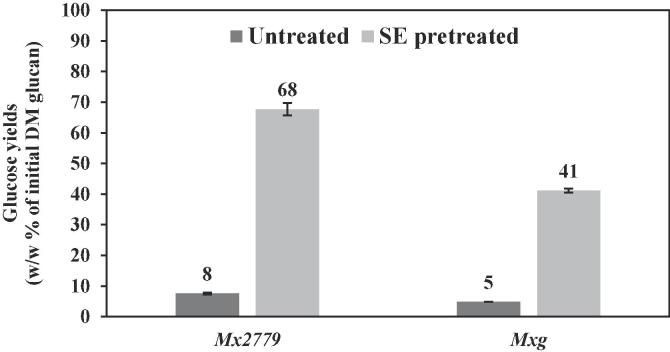
Enzymatic hydrolysis with Cellic® CTec2 of untreated and SE pretreated solids obtained from *Miscanthus* under SE pretreatment (200 °C; 15 bar; 10 min). Data are means ± standard error (n ≥ 2).

As the xylan component is partly solubilised into the hydrolysate during SE pretreatment, the remaining pretreated solids represent a fraction rich in glucan and lignin that can be further converted into bioethanol and biochemicals. As can be deduced from the glucose yields obtained after enzymatic hydrolysis over 72 h with Cellic® CTec2, SE pretreatment improved the digestibility performance of the pretreated biomass and glucan available to cellulases as compared to untreated material by 8 to 9-fold (Fig. 3). For *Mx2779* and *Mxg*, the glucose yields of the untreated material were only 8 and 5 % (w/w of initial glucan), respectively. Whereas following SE pretreatment, glucose yields of the pretreated material increased to ~70 and 40 % w/w, respectively (Fig. 3). These findings suggest that xylan removal and perhaps de-acetylation of xylan by SE pretreatment are amongst the factors that increase the accessibility of glucan in the pretreated residual solids to cellulolytic enzymes (Oliveira et al., 2013, Zhang et al., 2018). While the SE conditions were similar for *Mx2779* and *Mxg*, the different glucose yields from the two pretreated materials were suggestive of variable biomass recalcitrance between *Miscanthus* hybrids. Even though delignification was limited in the SE pretreated biomass, it was ~5-fold higher for *Mxg* (~20 %) compared to *Mx2779* (~4 %) (Table 2a), suggesting that delignification *per se* did not necessarily render the residual solids more susceptible to enzymatic attack (Fig. 3). However, it could be related to compositional and structural changes to biomass as a result of SE pretreatment, including rearrangements in the structure of glucan or lignin fragmentation and recondensation on pretreated solid surfaces, as well as the nature of the lignocellulosic matrix that lignin is present in (Oliveira et al., 2013, Pu et al., 2015). Hence, delignification and lignin transformations/relocation that occurs during SE pretreatment and their effects on biomass recalcitrance represent factors to elucidate (Pu et al., 2015).

### 3.6 Mass balance

**Fig. 4:**
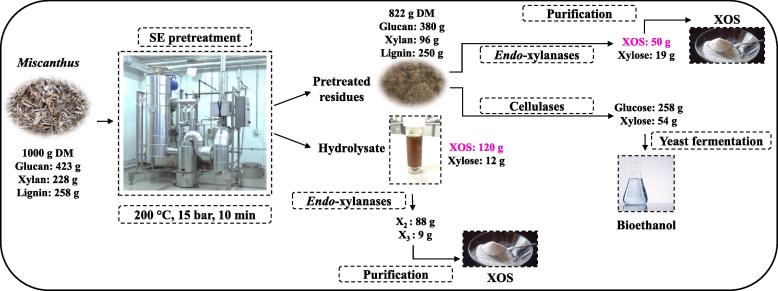
Overall mass balance of *Mx2779* under SE pretreatment and subsequent enzymatic hydrolysis of hydrolysate and residual solids. DM, dry matter; XOS, *xylo*-oligosaccharides.

An overall mass balance of the SE process and enzymatic hydrolysis steps applied to *Mx2779* is summarised in Fig. 4. Based on 1 kg of DM *Miscanthus* biomass, ~900 g (~90 %) of the original untreated solids were recovered after the pre-soaking step (Fig. 4). This is due to loss of the fine particles and biomass during the crudely conducted removal of excess liquid through a muslin cloth prior to SE pretreatment. For ~900 g DM of *Mx2779* input into the SE process (200 °C; 15 bar; 10 min), ~90 % was obtained as solid fraction (~380 g glucan, ~96 g xylan and ~250 g lignin) and the remaining ~10 % was dissolved into the hydrolysate mostly comprising of solubilised XOS (~120 g) (Fig. 4). Enzymatic hydrolysis after only 4 h of the SE hydrolysate with commercially available Novozymes *endo*-xylanases improved xylobiose quantities from ~12 to 88 g (Fig. 4), demonstrating that ~40 % from *Mx2779* and ~44 % (w/w of initial xylan) from *Mxg* could be converted into xylobiose. This is of particular industrial interest as the value-added compound xylobiose exhibits the strongest prebiotic activity amongst the XOS compounds and a higher sweetness potency than sucrose (Moura et al., 2007, Park et al., 2017). The less accessible ~40 % (w/w of initial xylan) remaining in the pretreated residual solids was also enzymatically released as XOS with the commercial Novozymes *endo*-xylanases NS22083 (Fig. 4). Hence, the overall process achieved a xylan (228 g) to XOS (170 g) conversion rate of ~75 %. To recover the maximum amount of XOS for an optimised biorefinery, the possibility to directly treat the whole SE slurry with *endo*-xylanases, prior to separating the slurry into SE pretreated solids and hydrolysate, as well as the potential to lower the enzyme dosage, requires further investigation. Furthermore, the glucan rich pretreated solids from *Mx2779* was subjected to 72 h enzymatic hydrolysis for initial investigation with a low solids loading (1 % w/v) and high dosage of Cellic® CTec2 (30 % g enzyme/g glucan) to indicate maximum enzymatically accessible glucan content, rather than a high solids loading and low dosage which should provide a target for commercially feasible glucan hydrolysis. We noted ~70 % of the glucan was released as glucose (equivalent to ~258 g of glucan) (Fig. 4), which can be further processed to bioethanol or platform chemicals via fermentation. Future testings will encompass the effects of shorter hydrolysis time, total solids loadings, glucan conversion and enzyme trial dosage levels. The post-enzymatic hydrolysis solids left behind were not quantified, although it is anticipated that this solid fraction should contain the remaining ~250 g of lignin that could be further used to produce lignin-based materials or combusted to contribute towards energy requirements within the proposed biorefinery process.

## 4 Conclusions

Pilot-scale production of XOS and fermentable sugars from *Miscanthus* was investigated using steam explosion (SE) pretreatment. Under the SE conditions studied (200 °C; 15 bar; 10 min), XOS yields up to 52 % (w/w of initial xylan) were obtained. The main effect of commercial *endo*-xylanases on the XOS-rich hydrolysate was the production of xylobiose (380–500 g/kg of initial xylan), a low-DP XOS having the highest pre-biotic potential. SE pretreatment also improved the cellulolytic hydrolysis of pretreated solids to increase the production of fermentable sugars by 8 to 9-fold. In view of an integrated biorefinery, SE represents a prospective pretreatment technology for the production of XOS and fermentable sugars from *Miscanthus*.

## Declaration of Competing Interest

The authors declare that they have no known competing financial interests or personal relationships that could have appeared to influence the work reported in this paper.
