# Supplementary material for: Pilot-scale production of xylo-oligosaccharides and fermentable sugars from Miscanthus using steam explosion pretreatment
Source: Bioresour Technol. 2020 Jan;296:122285. doi: 10.1016/j.biortech.2019.122285 (PMC6920740; doi:10.1016/j.biortech.2019.122285)
Supplement: Supplementary data 1 [file mmc1.pptx]

## Slide 1
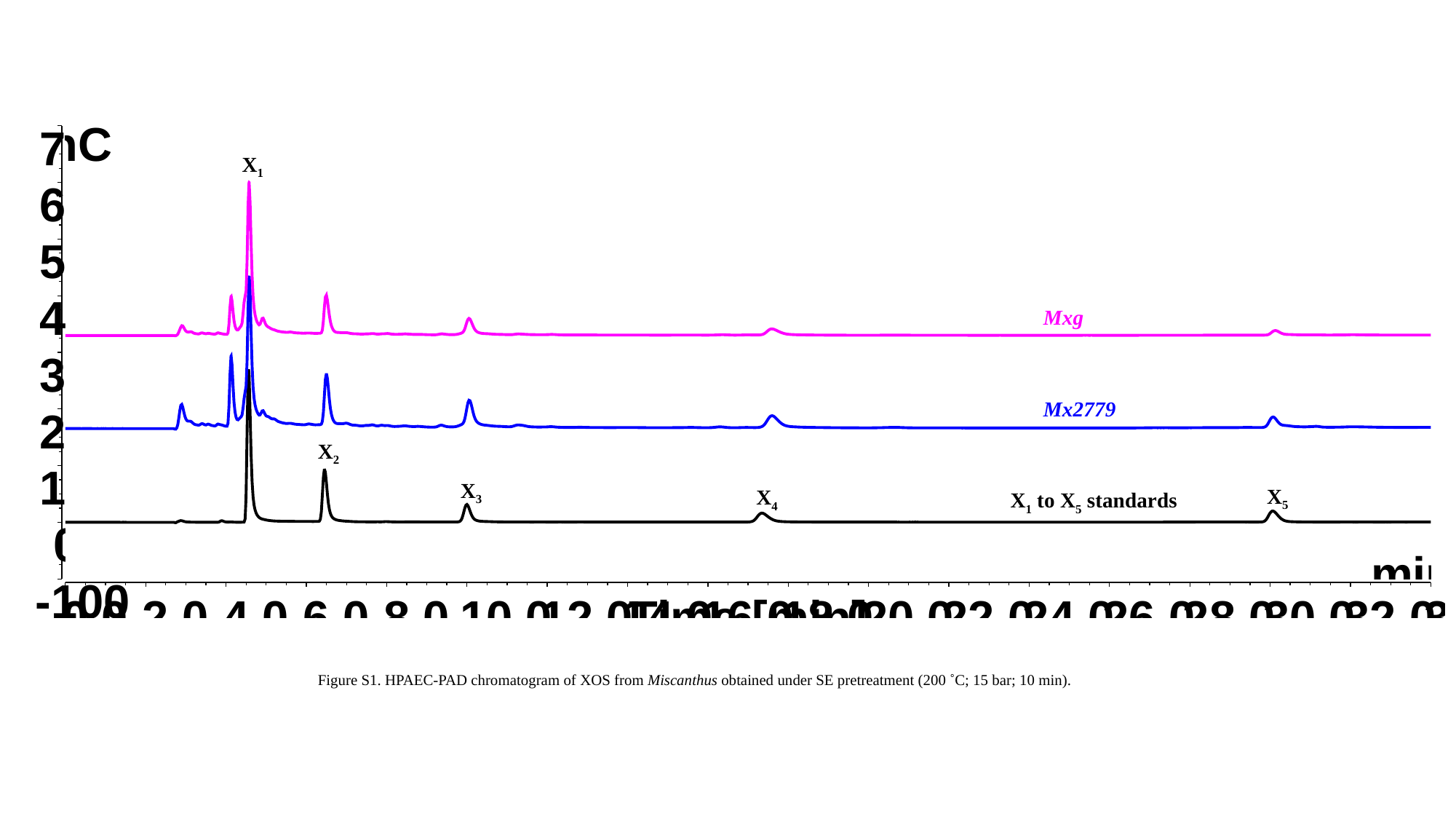

X1
Mxg
Mx2779
X2
X3
X5
X4
X1 to X5 standards
Figure S1. HPAEC-PAD chromatogram of XOS from Miscanthus obtained under SE pretreatment (200 ˚C; 15 bar; 10 min).

## Slide 2
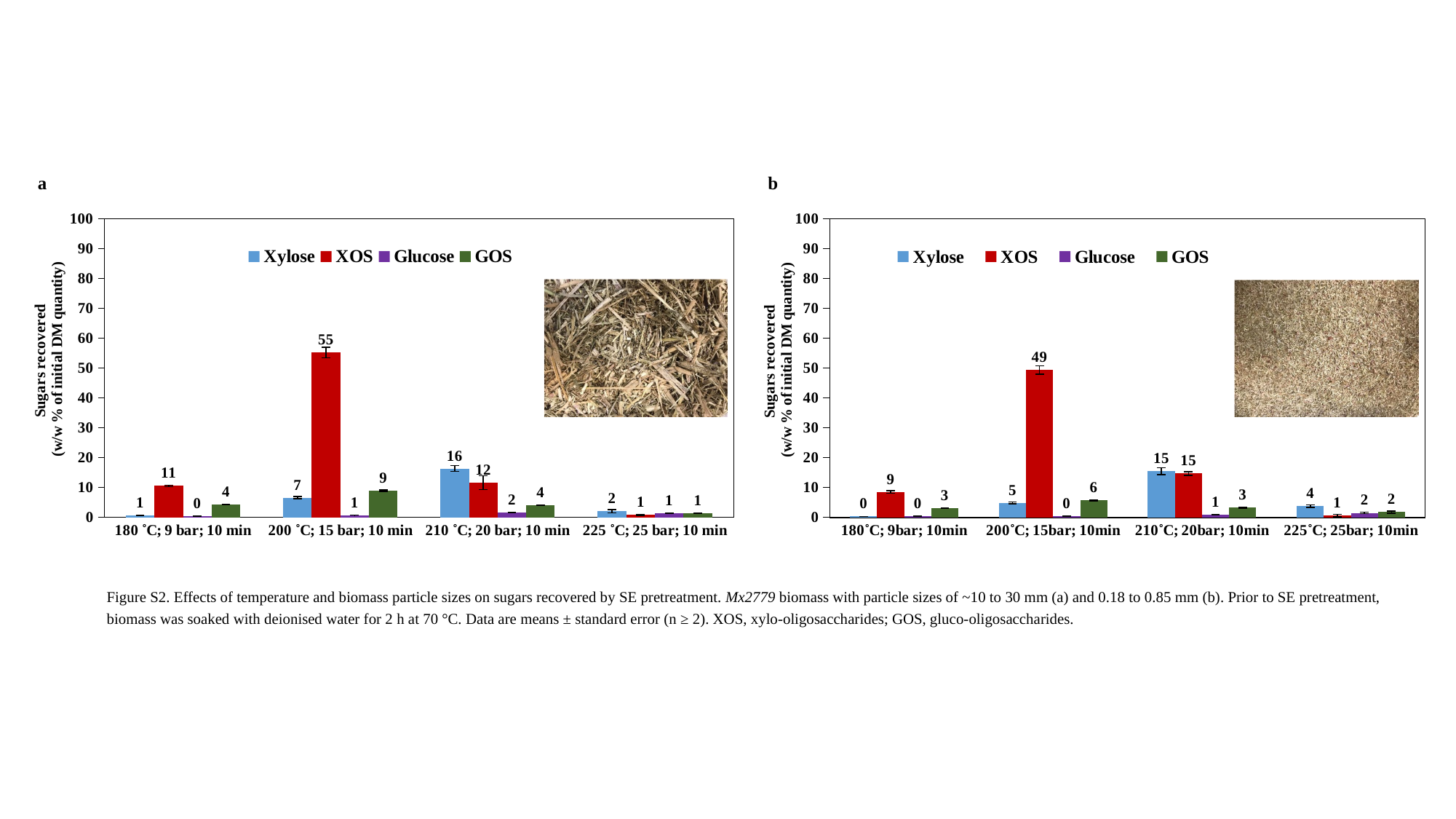

a
### Chart
| Category | | | | |
|---|---|---|---|---|
| 180 ˚C; 9 bar; 10 min | 0.6108928960396038 | 10.523086502549905 | 0.43152686085572844 | 4.297060228136929 |
| 200 ˚C; 15 bar; 10 min | 6.565916153010235 | 55.164951389850444 | 0.7331005075764614 | 8.895820664631156 |
| 210 ˚C; 20 bar; 10 min | 16.314268805723763 | 11.620508858002978 | 1.571402675557064 | 4.064278099138042 |
| 225 ˚C; 25 bar; 10 min | 2.0112505967896377 | 0.7840193855471387 | 1.3215541076437294 | 1.4325448892372012 |
b
### Chart
| Category | | | | |
|---|---|---|---|---|
| 180˚C; 9bar; 10min | 0.3259874556213017 | 8.545123274776266 | 0.35729500603791814 | 3.156643345494638 |
| 200˚C; 15bar; 10min | 4.894804622171565 | 49.35686994823 | 0.4748775076152887 | 5.755556519684618 |
| 210˚C; 20bar; 10min | 15.468477129940677 | 14.689714259193872 | 0.8250335444329175 | 3.285802941465017 |
| 225˚C; 25bar; 10min | 3.7707850551890774 | 0.5779721544193052 | 1.516083640437175 | 1.7776854287546016 |
Figure S2. Effects of temperature and biomass particle sizes on sugars recovered by SE pretreatment. Mx2779 biomass with particle sizes of ~10 to 30 mm (a) and 0.18 to 0.85 mm (b). Prior to SE pretreatment, biomass was soaked with deionised water for 2 h at 70 °C. Data are means ± standard error (n ≥ 2). XOS, xylo-oligosaccharides; GOS, gluco-oligosaccharides.

## Slide 3
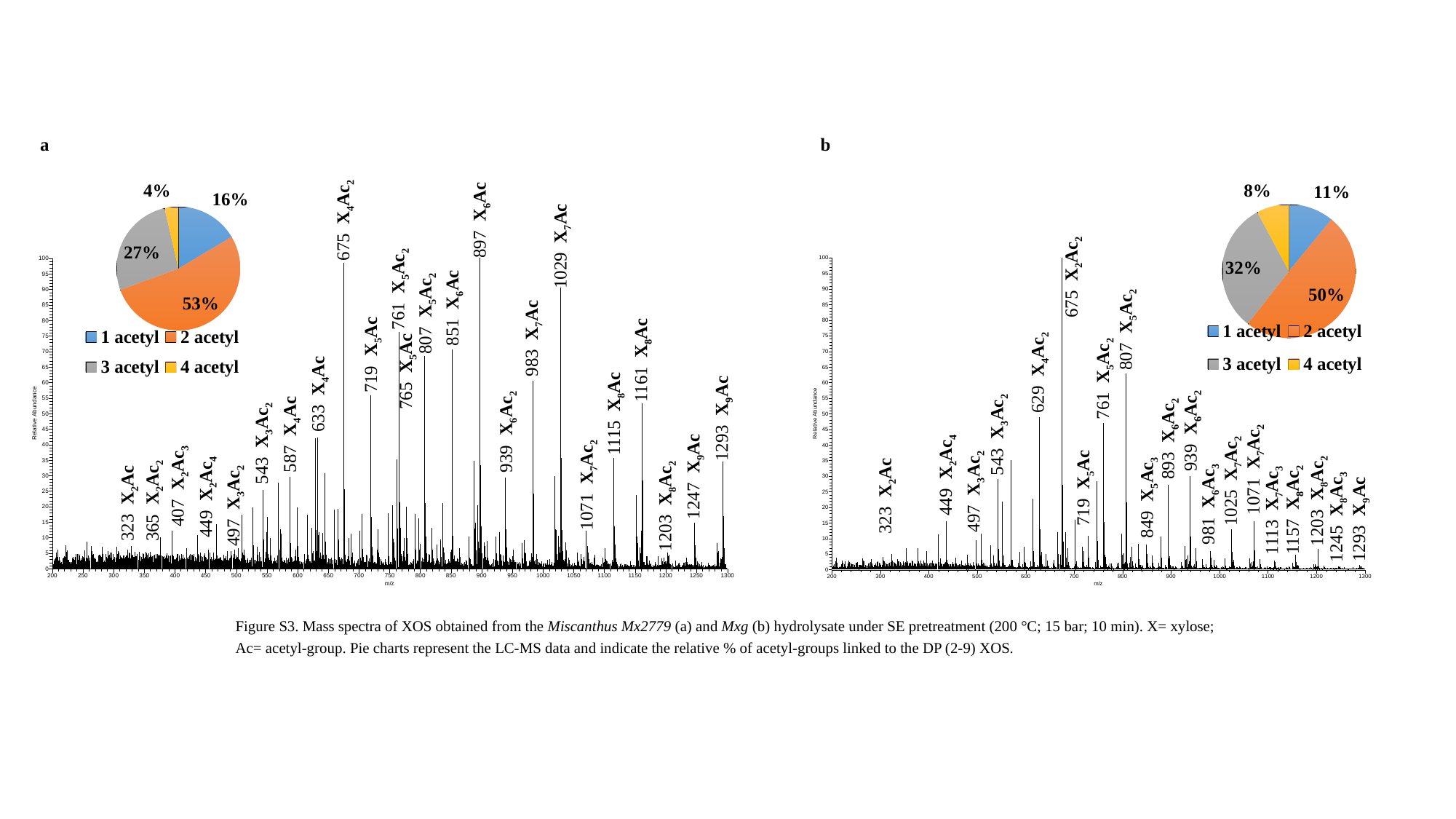

a
b
675 X4Ac2
897 X6Ac
### Chart
| Category | |
|---|---|
| 1 acetyl | 16.469789287875734 |
| 2 acetyl | 52.94844724631833 |
| 3 acetyl | 26.989803308452696 |
| 4 acetyl | 3.591977304821464 |1029 X7Ac
761 X5Ac2
851 X6Ac
983 X7Ac
765 X5Ac
1161 X8Ac
807 X5Ac2
719 X5Ac
633 X4Ac
939 X6Ac2
1115 X8Ac
1293 X9Ac
587 X4Ac
543 X3Ac2
1247 X9Ac
1071 X7Ac2
1203 X8Ac2
407 X2Ac3
449 X2Ac4
323 X2Ac
365 X2Ac2
497 X3Ac2
### Chart
| Category | |
|---|---|
| 1 acetyl | 10.954126876479418 |
| 2 acetyl | 49.608118598312 |
| 3 acetyl | 31.588113872931 |
| 4 acetyl | 7.849645246397991 |675 X2Ac2
807 X5Ac2
761 X5Ac2
629 X4Ac2
939 X6Ac2
543 X3Ac2
893 X6Ac2
1025 X7Ac2
1071 X7Ac2
497 X3Ac2
719 X5Ac
1203 X8Ac2
449 X2Ac4
849 X5Ac3
1113 X7Ac3
1157 X8Ac2
323 X2Ac
981 X6Ac3
1245 X8Ac3
1293 X9Ac
Figure S3. Mass spectra of XOS obtained from the Miscanthus Mx2779 (a) and Mxg (b) hydrolysate under SE pretreatment (200 °C; 15 bar; 10 min). X= xylose; Ac= acetyl-group. Pie charts represent the LC-MS data and indicate the relative % of acetyl-groups linked to the DP (2-9) XOS.

## Slide 4
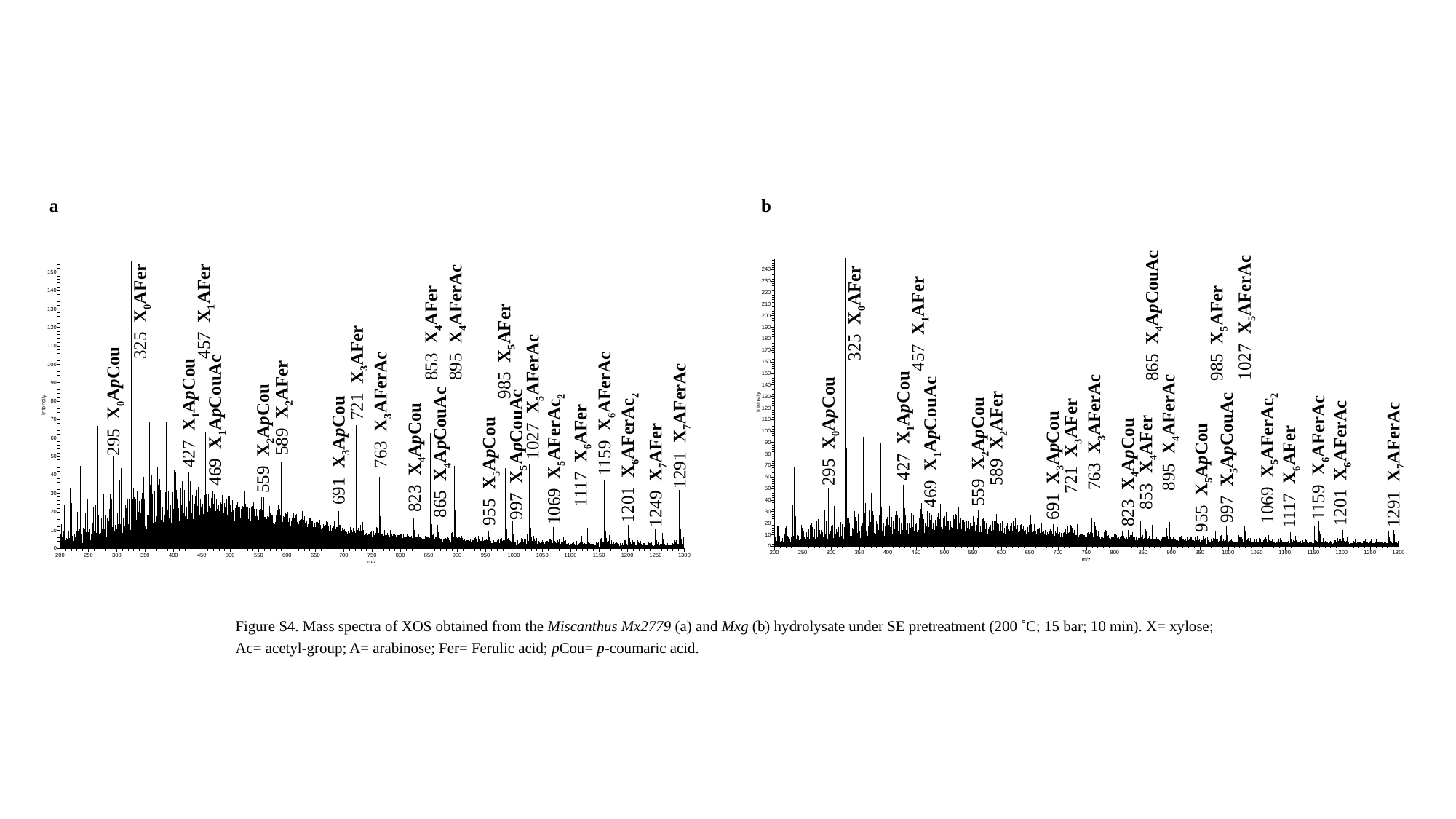

a
b
325 X0AFer
985 X5AFer
853 X4AFer
457 X1AFer
895 X4AFerAc
427 X1ApCou
721 X3AFer
1027 X5AFerAc
589 X2AFer
865 X4ApCouAc
1069 X5AFerAc2
295 X0ApCou
763 X3AFerAc
1159 X6AFerAc
691 X3ApCou
559 X2ApCou
1117 X6AFer
1291 X7AFerAc
823 X4ApCou
469 X1ApCouAc
1201 X6AFerAc2
1249 X7AFer
997 X5ApCouAc
955 X5ApCou
985 X5AFer
1027 X5AFerAc
865 X4ApCouAc
325 X0AFer
457 X1AFer
469 X1ApCouAc
427 X1ApCou
589 X2AFer
1159 X6AFerAc
763 X3AFerAc
895 X4AFerAc
1201 X6AFerAc
955 X5ApCou
1117 X6AFer
295 X0ApCou
997 X5ApCouAc
1291 X7AFerAc
1069 X5AFerAc2
853 X4AFer
691 X3ApCou
823 X4ApCou
559 X2ApCou
721 X3AFer
Figure S4. Mass spectra of XOS obtained from the Miscanthus Mx2779 (a) and Mxg (b) hydrolysate under SE pretreatment (200 ˚C; 15 bar; 10 min). X= xylose; Ac= acetyl-group; A= arabinose; Fer= Ferulic acid; pCou= p-coumaric acid.

## Slide 5
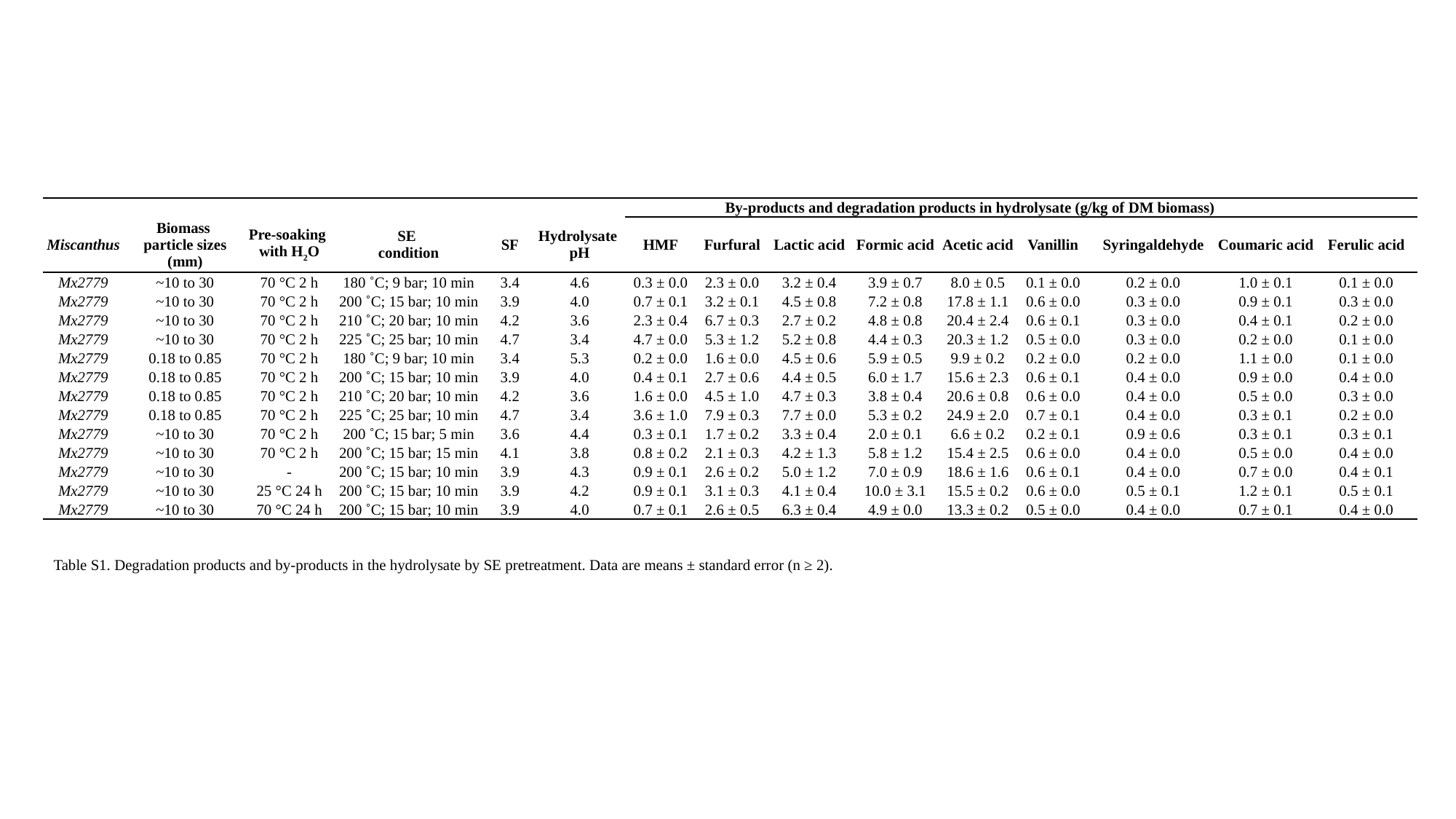

| | | | | | | By-products and degradation products in hydrolysate (g/kg of DM biomass) | | | | | | | | |
| --- | --- | --- | --- | --- | --- | --- | --- | --- | --- | --- | --- | --- | --- | --- |
| Miscanthus | Biomass particle sizes (mm) | Pre-soaking with H2O | SE condition | SF | Hydrolysate pH | HMF | Furfural | Lactic acid | Formic acid | Acetic acid | Vanillin | Syringaldehyde | Coumaric acid | Ferulic acid |
| Mx2779 | ~10 to 30 | 70 °C 2 h | 180 ˚C; 9 bar; 10 min | 3.4 | 4.6 | 0.3 ± 0.0 | 2.3 ± 0.0 | 3.2 ± 0.4 | 3.9 ± 0.7 | 8.0 ± 0.5 | 0.1 ± 0.0 | 0.2 ± 0.0 | 1.0 ± 0.1 | 0.1 ± 0.0 |
| Mx2779 | ~10 to 30 | 70 °C 2 h | 200 ˚C; 15 bar; 10 min | 3.9 | 4.0 | 0.7 ± 0.1 | 3.2 ± 0.1 | 4.5 ± 0.8 | 7.2 ± 0.8 | 17.8 ± 1.1 | 0.6 ± 0.0 | 0.3 ± 0.0 | 0.9 ± 0.1 | 0.3 ± 0.0 |
| Mx2779 | ~10 to 30 | 70 °C 2 h | 210 ˚C; 20 bar; 10 min | 4.2 | 3.6 | 2.3 ± 0.4 | 6.7 ± 0.3 | 2.7 ± 0.2 | 4.8 ± 0.8 | 20.4 ± 2.4 | 0.6 ± 0.1 | 0.3 ± 0.0 | 0.4 ± 0.1 | 0.2 ± 0.0 |
| Mx2779 | ~10 to 30 | 70 °C 2 h | 225 ˚C; 25 bar; 10 min | 4.7 | 3.4 | 4.7 ± 0.0 | 5.3 ± 1.2 | 5.2 ± 0.8 | 4.4 ± 0.3 | 20.3 ± 1.2 | 0.5 ± 0.0 | 0.3 ± 0.0 | 0.2 ± 0.0 | 0.1 ± 0.0 |
| Mx2779 | 0.18 to 0.85 | 70 °C 2 h | 180 ˚C; 9 bar; 10 min | 3.4 | 5.3 | 0.2 ± 0.0 | 1.6 ± 0.0 | 4.5 ± 0.6 | 5.9 ± 0.5 | 9.9 ± 0.2 | 0.2 ± 0.0 | 0.2 ± 0.0 | 1.1 ± 0.0 | 0.1 ± 0.0 |
| Mx2779 | 0.18 to 0.85 | 70 °C 2 h | 200 ˚C; 15 bar; 10 min | 3.9 | 4.0 | 0.4 ± 0.1 | 2.7 ± 0.6 | 4.4 ± 0.5 | 6.0 ± 1.7 | 15.6 ± 2.3 | 0.6 ± 0.1 | 0.4 ± 0.0 | 0.9 ± 0.0 | 0.4 ± 0.0 |
| Mx2779 | 0.18 to 0.85 | 70 °C 2 h | 210 ˚C; 20 bar; 10 min | 4.2 | 3.6 | 1.6 ± 0.0 | 4.5 ± 1.0 | 4.7 ± 0.3 | 3.8 ± 0.4 | 20.6 ± 0.8 | 0.6 ± 0.0 | 0.4 ± 0.0 | 0.5 ± 0.0 | 0.3 ± 0.0 |
| Mx2779 | 0.18 to 0.85 | 70 °C 2 h | 225 ˚C; 25 bar; 10 min | 4.7 | 3.4 | 3.6 ± 1.0 | 7.9 ± 0.3 | 7.7 ± 0.0 | 5.3 ± 0.2 | 24.9 ± 2.0 | 0.7 ± 0.1 | 0.4 ± 0.0 | 0.3 ± 0.1 | 0.2 ± 0.0 |
| Mx2779 | ~10 to 30 | 70 °C 2 h | 200 ˚C; 15 bar; 5 min | 3.6 | 4.4 | 0.3 ± 0.1 | 1.7 ± 0.2 | 3.3 ± 0.4 | 2.0 ± 0.1 | 6.6 ± 0.2 | 0.2 ± 0.1 | 0.9 ± 0.6 | 0.3 ± 0.1 | 0.3 ± 0.1 |
| Mx2779 | ~10 to 30 | 70 °C 2 h | 200 ˚C; 15 bar; 15 min | 4.1 | 3.8 | 0.8 ± 0.2 | 2.1 ± 0.3 | 4.2 ± 1.3 | 5.8 ± 1.2 | 15.4 ± 2.5 | 0.6 ± 0.0 | 0.4 ± 0.0 | 0.5 ± 0.0 | 0.4 ± 0.0 |
| Mx2779 | ~10 to 30 | - | 200 ˚C; 15 bar; 10 min | 3.9 | 4.3 | 0.9 ± 0.1 | 2.6 ± 0.2 | 5.0 ± 1.2 | 7.0 ± 0.9 | 18.6 ± 1.6 | 0.6 ± 0.1 | 0.4 ± 0.0 | 0.7 ± 0.0 | 0.4 ± 0.1 |
| Mx2779 | ~10 to 30 | 25 °C 24 h | 200 ˚C; 15 bar; 10 min | 3.9 | 4.2 | 0.9 ± 0.1 | 3.1 ± 0.3 | 4.1 ± 0.4 | 10.0 ± 3.1 | 15.5 ± 0.2 | 0.6 ± 0.0 | 0.5 ± 0.1 | 1.2 ± 0.1 | 0.5 ± 0.1 |
| Mx2779 | ~10 to 30 | 70 °C 24 h | 200 ˚C; 15 bar; 10 min | 3.9 | 4.0 | 0.7 ± 0.1 | 2.6 ± 0.5 | 6.3 ± 0.4 | 4.9 ± 0.0 | 13.3 ± 0.2 | 0.5 ± 0.0 | 0.4 ± 0.0 | 0.7 ± 0.1 | 0.4 ± 0.0 |
Table S1. Degradation products and by-products in the hydrolysate by SE pretreatment. Data are means ± standard error (n ≥ 2).
